# Modelling the effects of booster dose vaccination schedules and recommendations for public health immunization programs: the case of *Haemophilus influenzae* serotype b

**DOI:** 10.1186/s12889-017-4714-9

**Published:** 2017-09-13

**Authors:** Nadia A. Charania, Seyed M. Moghadas

**Affiliations:** 1 Department of Public Health, Auckland University of Technology, 640 Great South Road, Manukau, Auckland, 2025 New Zealand; 2 Agent-Based Modelling Laboratory, York University, 4700 Keele Street, Toronto, ON M3J 1P3 Canada

**Keywords:** *Haemophilus influenzae*, Stochastic simulations, Epidemic modelling, Booster vaccination, Vaccine policy, Public health

## Abstract

**Background:**

*Haemophilus influenzae* serotype b (Hib) has yet to be eliminated despite the implementation of routine infant immunization programs. There is no consensus regarding the number of primary vaccine doses and an optimal schedule for the booster dose. We sought to evaluate the effect of a booster dose after receiving the primary series on the long-term disease incidence.

**Methods:**

A stochastic model of Hib transmission dynamics was constructed to compare the long-term impact of a booster vaccination and different booster schedules after receiving the primary series on the incidence of carriage and symptomatic disease. We parameterized the model with available estimates for the efficacy of Hib conjugate vaccine and durations of both vaccine-induced and naturally acquired immunity.

**Results:**

We found that administering a booster dose substantially reduced the population burden of Hib disease compared to the scenario of only receiving the primary series. Comparing the schedules, the incidence of carriage for a 2-year delay (on average) in booster vaccination was comparable or lower than that observed for the scenario of booster dose within 1 year after primary series. The temporal reduction of symptomatic disease was similar in the two booster schedules, suggesting no superiority of one schedule over the other in terms of reducing the incidence of symptomatic disease.

**Conclusions:**

The findings underscore the importance of a booster vaccination for continued decline of Hib incidence. When the primary series provides a high level of protection temporarily, delaying the booster dose (still within the average duration of protection conferred by the primary series) may be beneficial to maintain longer-term protection levels and decelerate the decline of herd immunity in the population.

**Electronic supplementary material:**

The online version of this article (10.1186/s12889-017-4714-9) contains supplementary material, which is available to authorized users.

## Background

*Haemophilus influenzae* serotype b (Hib), a gram-negative coccobacillus, was one of the leading causes of bacterial meningitis and pneumonia in children under 5 years of age and immunocompromized adults in the pre-Hib vaccine era [1]. The incidence of Hib has dramatically decreased since the introduction of Hib conjugate vaccines in the early 1990s [2]. All Hib vaccines currently licensed for use are conjugated and available as a monovalent vaccine or as a combination vaccine with other antigens [2]. The uptake of these vaccines in childhood immunization programs of many countries has increased globally, largely driven by the World Health Organization (WHO) recommendations for primary and booster doses [2]. In a number of countries, the routine use of Hib conjugate vaccines has provided direct protection for vaccinated children and induced herd immunity, thereby providing indirect protection to the entire population [2, 3].

Despite the introduction of routine infant immunization programs, Hib infection has yet to be eliminated and instances of Hib resurgence have occurred [4–6]. A number of explicators have been proposed for the increase in the incidence of Hib in recent years, including the use of combination vaccines containing acellular pertussis that have lower immunogenicity compared to monovalent Hib conjugate vaccines [7–9]. Recent studies have also reported that Hib antibody concentrations wane after primary series immunization, highlighting the role of a booster dose to maintain adequate immune protection levels [10–12]. For instance, data from the United Kingdom suggested that although implementing an infant immunization program without a booster dose initially resulted in decreased rates of invasive Hib disease, these rates later increased [13, 14] and the introduction of a booster campaign helped re-establish herd immunity [10]. Furthermore, a modelling study of Hib disease in the United States during a period of vaccine shortage indicated that deferral of the Hib booster dose could result in Hib resurgence 3 years after the start of the vaccine shortage [6]. Moreover, while the importance of being age-appropriately vaccinated to control the transmission of vaccine-preventable diseases has been noted, parental vaccine hesitancy, deferral and refusal have played a large role in suboptimal compliance with recommended childhood immunization schedules [15, 16]. A recent study in the United States reported that during 2002–2009, more than half of Hib cases occurred among children who were eligible for vaccination but were either behind schedule or completely unvaccinated [17].

The WHO recommends Hib vaccine schedules comprised of either 3 primary doses without a booster or 2 to 3 primary doses plus a booster given at least 6 months after completing the primary series [18]. However, there is no consensus regarding an optimal Hib vaccination schedule and booster schedules vary around the world [19]. Many uncertainties remain, particularly in terms of the optimal number of doses offered and the dosing interval between primary and booster vaccination [19]. Given the accumulating evidence regarding the role of a primary series plus a booster dose for curtailing Hib disease, we developed a stochastic model of Hib transmission dynamics to evaluate the impact of a booster vaccination with different schedules on the long-term disease incidence in the population. This evaluation is particularly important for devising optimal vaccination strategies that can result in disease elimination.

## Methods

**Fig. 1:**
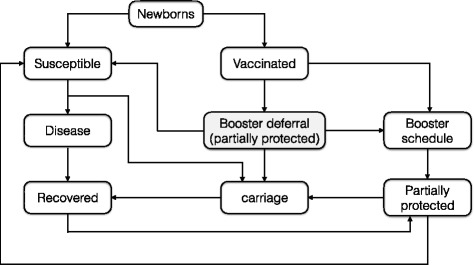
Model diagram for transition between epidemiological compartments in the population

To develop the epidemiological model, we made some realistic assumptions as validated in biological studies [6, 20]. We divided the population into classes of susceptible individuals (*S*) with no prior exposure to infection; exposed individuals who are infected but not yet infectious (*L*); infectious individuals who are subclinical (*C*) referred to as carriage and transmit the disease without developing clinical manifestations; and infectious individuals with clinical manifestations of the disease (*I*). To include vaccination, we considered other classes of individuals depending on their immune protection level induced by vaccine or natural infection. We denote infants who receive the primary immunization series within the first year of their life by *V*_*n*_. We assumed that primary vaccination provides only partial protection [21, 22], and included the class of *V*_*p*_ for individuals who have received the primary immunization series and are still partially protected in the second year of their life. Individuals with primary vaccination are eligible to receive a booster dose, which provides full protection for a certain period of time, and we denote the class of individuals who receive a booster dose in the second year of their life by *V*_*b*_. The class of those who defer booster vaccination is denoted by *D*. If vaccinated with the deferred booster dose, individuals will acquire full protection. The immunity induced by booster vaccination wanes over time, and individuals may enter the class *V*_*bp*_, having only partial protection. We assumed that immune protection in individuals who do not receive booster wanes over time below protective levels, making them susceptible to infection again. We also assumed that individuals with partial protection may become infected if transmission takes place, but develop carriage [6]. Recovery from carriage or symptomatic disease is assumed to provide full protection for a certain period of time, followed by a partial protection era. Figure 1 represents a schematic model diagram for transitions between different classes of individuals. Based on the assumption of homogeneously mixing population, the model can be expressed by a system of differential equations using a proportional incidence of infection (see Additional file 1).

### The basic reproduction number

An important epidemiological parameter in the study of disease dynamics is the basic reproduction number, commonly denoted by *R*_0_, which is defined as the expected number of secondary infections produced by a single infectious individual in an entirely susceptible population during his or her infectious period [23]. If *R*_0_ < 1, then on average an infected individual produces less than one new infection, and therefore the number of infections cannot grow. On the other hand, if *R*_0_ > 1, then each infected individual produces, on average, more than one new infection, and the disease may cause an outbreak. Using an individual tracing method [24], one can obtain the number of secondary infections in our model in the absence of any control measures (such as vaccination) by:

$$ {R}_0=\frac{q\delta\theta\beta}{\left(\mu +\theta \right)\left(\mu +{\gamma}_1\right)}+\frac{\left(1-q\right)\theta\beta}{\left(\mu +\theta \right)\left(\mu +{\gamma}_2\right)} $$

Parameters in the expression of *R*_0_ are described in Table 1.

**Table 1:** Description of model parameters with their values or ranges used for the stochastic simulations from the published literature

Parameter	Description	Value (range)
***R***_0_	basic reproduction number	1.4 (1.3 − 1.5)
***β***	baseline transmission rate of infection	variable
***p***	infant primary vaccination coverage	0.9 (0 − 1)
***α***	fraction of individuals who defer booster vaccination	variable (0 − 100%)
***r***	fraction of individuals who receive booster with deferral	100 % (0 − 100%)
***η***	reduction of transmissibility during partial protection after primary vaccination	0.15 (0.1 − 0.2)
***π***	reduction of transmissibility during partial protection after natural infection or booster vaccination	0.5 (0.3 − 0.7)
***δ***	reduction of transmissibility during carriage	0.5 (0.3 − 0.7)
1/***σ***	age for completion of primary vaccination	0.5 year
1/***ϕ***	duration of time for receiving booster after primary vaccine	1 (1 − 4) years
1/***κ***	average duration of partial protection without booster	2 (1 − 3) years
1/***ξ***	average duration of full protection following booster	4 (2 − 6) years
1/***τ***	average duration of partial protection after natural infection or booster	6 (4 − 8) years
1/***ε***	average duration of full protection following infection	3 (2 − 4) years
1/***θ***	average duration of latency	2 days
1/***γ***_1_	average duration of carriage	60 (14 − 120) days
1/***γ***_2_	average duration of communicability for symptomatic infection	2 days
1/***μ***	average lifespan	70 years
***q***	fraction of infected individuals who undergo carriage	(0.6 − 0.9)

### Stochastic model implementation

We used a Markov Chain Monte Carlo method to simulate the stochastic model with a total population size of *N* = 100,000. To simulate the scenarios with vaccination, we parameterized the model with available estimates from the published literature (Table 1), and generated the initial conditions for seeding the model states using a 50-year warm-up simulation period for stabilization. The equilibrium of the system without vaccination was reached during 50 years of the warm-up period, which was initiated with 5 cases of carriage and one case of symptomatic disease. This equilibrium was used as the initial condition for simulating the scenarios with vaccination. We used the Gillespie direct algorithm to run stochastic simulations [25]. In this way, to estimate the transition time to the next event, we let *dt* = *ℓ*_1_/Δ, where *ℓ*_1_ is a random number drawn from the uniform distribution on the unit interval [0, 1], and Δ is equal to the sum of the rates for all possible events. We then ordered the events as an increasing fraction of Δ and generated another uniform number *ℓ*_2_ between 0 and 1 to determine the nature of the next event. We ran 1000 independent simulations to calculate the average of sample realizations of the stochastic process in each scenario.

### Model parameterization

A number of parameters were varied in our simulations to evaluate the effect of primary series, booster dose, and deferral of booster vaccination on the incidence of carriage and symptomatic disease over a 30-year period after the start of vaccination. The transmission parameter was calculated based on a given basic reproduction number, while fixing other parameters of the model. We assumed *R*_0_ = 1.4 in the range 1.3–1.5 estimated in studies of Hib [26], while lower (*R*_0_ = 1.04) [27, 28] and higher (*R*_0_ = 3.3) [29] reproduction numbers have also been reported. Using previous estimates for Hib in the context of conjugate vaccines, we parametrized the model for the duration of naturally acquired immunity following recovery from infection, and the duration of primary and booster vaccine-induced immunity, and partial protection [6, 30, 31]. Similar to estimates for Hib [30, 32], we considered the probability of carriage following primary infection in the range 0.6–0.9. It is assumed that infection in the form of carriage is 50% less infectious than symptomatic disease. Furthermore, susceptibility to encounter new infection during partial protection is assumed to be reduced by 50%. We considered a latent period of 2 days after colonization during which no transmission can occur [30]. The duration of carriage is unknown, but may range from several days to several weeks [6, 27, 28, 32]. We assumed an average infectious period of 60 days for carriage. The symptomatic infection is considered non-communicable within 24–48 h after starting effective antibiotic treatment [33]. Since individuals with symptomatic disease are likely to receive treatment, considering a delay of 1 day for start of treatment, we assumed an average infectious period of 2 days. Other parameters of the model are provided in Table 1.

### Simulation scenarios

The baseline scenario was simulated in the absence of vaccination to reach the system equilibrium. For vaccination scenarios, the coverage of primary series for infants within the first year of their life was fixed at 90%. We ran simulations with a booster dose and for two different scenarios in which the timing of the booster vaccination for those who received primary vaccination was varied. Deferral of booster vaccination corresponds to a scenario where the primary vaccinated individuals receive the booster dose beyond 2 years of age, and the duration of booster deferral was between 2 and 4 years of age.

## Results

### The effect of primary series vaccination

**Fig. 2:**
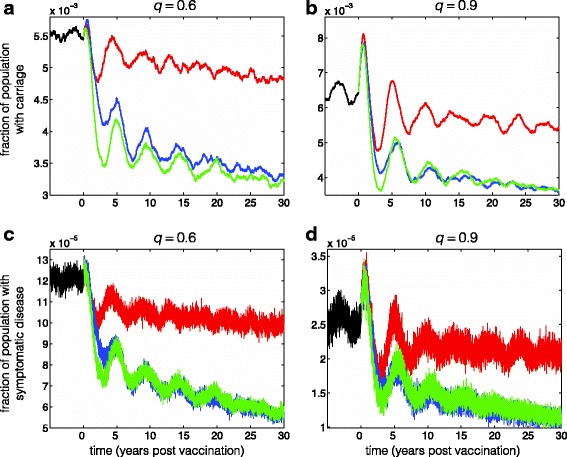
Fraction of carriage (**a**,**b**) and symptomatic disease (**c**,**d**) in the population over a 30-year period of simulations following the start of vaccination. The coverage of primary series vaccination was fixed at 90%. The parameter *q* represents the fraction of infected individuals who experience carriage. Black curves represent the equilibrium state of the system prior to the start of vaccination, at which the disease remains endemic in the population. Red curves show the scenario in which only primary vaccination is offered to infants within 6 months after birth. Blue curves represent the scenario in which primary vaccination of infants is combined with 100% coverage of the booster dose within 1 year after the primary series. Green curves show the scenario in which primary vaccination of infants is combined with 100% coverage of the booster dose between 2 and 4 years after the primary series

Figure 2 shows the time profiles of carriage and symptomatic disease for 30 years after the start of vaccination. Vaccination with only the primary series, achieving 90% coverage of infants within 6 months after birth (Fig. 2, red curves), leads to a damped oscillatory behaviour with temporal reduction in the incidence of both carriage and symptomatic disease. Since infection following primary series leads to carriage, we observed an initial increase in the number of cases at the start of vaccination. Furthermore, the immune protection induced by the primary series is assumed to be partially protective and wanes over time [6, 14, 34], and therefore an increase in the incidence of carriage is observed several years after the onset of the vaccination program. This leads to the rise of herd immunity in the population, which in turn reduces the incidence of infection as observed in the later decline of incidence for both carriage and symptomatic disease. The time profiles simulated here show that the disease still persists in the population with only primary series vaccination.

### The effect of booster dose vaccination

Considering a fixed coverage of 90% infant immunization for primary series vaccination, we implemented a single-dose booster within 1 year after the primary series. A booster dose with 100% coverage for primary vaccinated individuals substantially reduces the incidence of carriage and symptomatic disease (Fig. 2, blue curves) compared to the scenario of only primary series (Fig. 2, red curves).

### The effect of booster dose deferral

To explore the effect of booster deferral, we implemented the booster dose with 100% coverage between 2 and 4 years after the primary series (Fig. 2, green curves). This scenario corresponds to delaying the booster dose by 2 years (on average) for all primary vaccinated individuals. We found that the incidence of carriage over 30 years of simulations is comparable or lower than what is observed when a booster dose was offered within 1 year after the primary series (Fig. 2a,b, green curves). However, the temporal reduction of symptomatic disease was similar in the two booster schedules (Fig. 2c,d, green and blue curves), suggesting no superiority of the deferred booster dose over the schedule of booster within 1 year after the primary series in reducing the incidence of symptomatic disease.

## Discussion

In this study, we evaluated the effect of a booster vaccination with two different schedules following primary vaccination on the long-term disease incidence using stochastic model simulations based on parameter values estimated for Hib from the published literature.

### Booster dose vaccination

Consistent with previous observations [30], the results from the model presented herein indicate that infant immunization programs alone cannot eliminate the infection from the population even when a high coverage of the primary series is achieved. There is compelling evidence demonstrating the effectiveness of administering an infant primary series plus a booster dose to reduce the burden of Hib disease [10, 11]. Our results show that receiving a booster dose substantially reduced the incidence of carriage and symptomatic disease compared to the scenario of only receiving the primary series. In the case of Hib, previous studies show that the levels of clinical protection against disease wane over time after primary vaccination [34]; thus, individuals can become susceptible again and boosting may play a key role in maintaining herd immunity [12].

There are diverse Hib vaccination schedules worldwide which are reflective of several factors, including local epidemiology, vaccine composition (whether offered as a monovalent or combination vaccine), and the existing health services infrastructure and childhood immunization programs in different countries [2, 19]. While all low-income countries have adopted a 3-dose primary series, most high-income countries offer a 3-dose primary series plus a booster dose given at 11 months old or during the second year of life [12, 19]. However, there is no consensus about whether a booster dose is needed or not to sustain control of Hib disease [19]. For instance, waning immunity after the primary series has led some countries, such as the United Kingdom and Mexico, to introduce a booster dose after initially only recommending a primary series [9, 14, 35]. However, data from other countries (e.g., Kenya, The Gambia) indicate that a primary series without a booster dose has resulted in sustained reduction of Hib disease burden with no evidence of resurgence at this time [36, 37]. Although a number of studies suggest that a booster dose of Hib vaccine following infant immunization is not essential [17, 34, 36, 37], they also indicate that the levels of clinical protection against Hib disease wane over time after primary vaccination. While the rates of invasive Hib disease are reported to be declining in some countries with only primary series vaccination, the rates of carriage in the general population are not ascertained and the level of herd immunity remains unknown. Given the importance of carriage in the persistence of disease in the population [27] and the gradual loss of immune protection conferred by the primary series [6, 14, 34], the additional protective benefits of a booster dose should not be discounted in these settings. Overall, this highlights the importance of continuous surveillance to monitor changes in Hib incidence and carriage to guide optimal national vaccination policies.

### Booster dose timing

For the time interval between the primary series and booster dose, we observed that a 2-year (on average) delay in booster vaccination may lead to similar or higher reduction of carriage compared to the scenario of a booster dose within 1 year after primary series (Fig. 2a, b, blue and green curves). The timelines used in our simulations are consistent with reported estimates, indicating that Hib antibody levels induced by the infant primary vaccination series wane over 2–4 years from high to low in the absence of boosting [6, 14, 34]. While no difference in the outcomes of the two booster schedules was noted in terms of reducing the incidence of symptomatic diseases (Fig. 2c,d, blue and green curves), the potential for lower carriage rates suggest that the booster schedule with a 2-year deferral may be beneficial to maintain longer-term protection levels and decelerate the decline of herd immunity in the population.

Providing the booster dose to older children may be beneficial in terms of eliciting a stronger immune response. Previous literature has shown that levels of Hib antibody response and persistence correlate with the age at which the child is given the booster dose [38]. Comparing antibody responses to an anti-Hib booster dose among 3 age groups (6–11 months, 12–17 months, and 2–4 years), the study indicates that Hib antibody concentrations increased as the age at boosting increased, with a 6.1 fold difference noted between the youngest and oldest age groups [38]. After boosting, an age-independent decline in antibody titres occurred, indicating that the higher concentrations in the oldest age group (2–4 years) translated into greater persistence of long-term protection [38]. Evidence from the United Kingdom suggested that a catch-up vaccination program of children up to 4 years old in tandem with an infant vaccination program may have played a role in reducing the incidence of Hib [34]. It has also been demonstrated that offering an infant primary series plus a pre-school booster dose confers better long-term protection against Hib carriage compared to the program for an infant primary series plus a 12-month routine booster dose [39]. Moreover, adults generally lack adequate seroprotection since the booster dose only confers a relatively short duration of protection compared to the average lifetime, and opportunities for natural boosting is almost absent due to the reduced Hib exposure in the vaccine era [39]. The vulnerability of adults to Hib disease, especially those who are immunocompromized or have underlying co-morbidities, underscores the importance of reducing incidence rates, and particularly carriage amongst pre-school children and adolescents [27, 39]. Thus, receiving a booster dose at the pre-school age may provide more sustained protection against Hib, and offer some additional indirect protection to adults [39].

### Implications for public health immunization programs

Our results have important implications for public health immunization programs. First, they indicate the protective effect and additional benefits of reduced Hib incidence provided by vaccination programs that include a primary series plus a booster dose. Second, a booster schedule with a 2-year (on average) delay may be beneficial for reducing the incidence of carriage, although it provides no advantage over the scenario in which the booster is given within 1 year after primary series in reducing the incidence of symptomatic disease. However, the potential for transmission between young children (who are at higher risk of symptomatic disease) and adults (who are at higher risk of carriage) underscores the importance of reducing carriage rates to maintain the protective effects of vaccination programs [27]. Given these considerations, our study should stimulate a nexus of constructive critical dialogue on optimal vaccination programs, particularly regarding the timing of a booster dose. This is especially relevant to immunization policies for new vaccines that are being developed for other diseases with similar characteristics; for example, *Haemophilus influenzae* serotype ‘a’ (Hia) for which a bivalent Hib-Hia conjugate vaccine may be a potential candidate [31, 40].

### Limitations

This study has several limitations that merit further investigation. We did not structure the model by age, but we understand that the incidence of Hib could be affected by contact patterns between different age groups, especially among young children [6, 27, 41]. We also note that antigens from several organisms other than Hib can induce cross-reactive antibodies to Hib capsular polysaccharide [42–44], and may therefore boost acquired immunity and increase the protection levels against Hib disease. In our model, we assumed that exposure to infection during partial protection (if infection occurs) leads to carriage, but not to the symptomatic disease. However, depending on the level of immune protection, symptomatic disease may still develop. For simulations, we calculated the transmission rates using the expression for the basic reproduction number, while fixing other parameters in this expression. However, disease transmission is greatly affected by population demographics, heterogeneity in contacts, contact tracing, access to healthcare resources, health statuses of individuals and immunization programs. Considering these factors will require more complex and data-driven models to include individual characteristics and behaviour, in addition to the effect of control and preventive measures in different age groups of the population. Despite these limitations, our findings demonstrate the importance of booster vaccination, and calls for future studies to investigate the optimal timing for booster vaccination based on the efficacy and duration of vaccine-induced protection for Hib and other vaccine-preventable diseases with similar characteristics.

## Conclusions

We used a stochastic model of Hib transmission dynamics to evaluate the effect of a booster vaccination with different schedules after receiving the primary series on the long-term disease incidence. In addition to demonstrating the important role that a booster dose can play in reducing disease burden, we compared the currently in practice booster schedule with a 2-year (on average) deferred booster schedule. The findings indicate that in instances where the primary series provides high levels of protection temporarily, delaying the booster dose (still within the average duration of protection conferred by the primary series) may be beneficial to further reduce the rates of carriage, without negatively affecting the rates of symptomatic disease.

## Additional file

Additional file 1: The model equations and sensitivity and uncertainty analyses. (PDF 511 kb)

## Abbreviations

Hib: *Haemophilus influenzae* serotype b
WHO: World Health Organization
