# Supplementary material for: Modelling the effects of booster dose vaccination schedules and recommendations for public health immunization programs: the case of Haemophilus influenzae serotype b
Source: BMC Public Health. 2017 Sep 13;17:705. doi: 10.1186/s12889-017-4714-9 (PMC5598080; doi:10.1186/s12889-017-4714-9)
Supplement: Additional file 1: — The model equations and sensitivity and uncertainty analyses. (PDF 511 kb) [file 12889_2017_4714_MOESM1_ESM.pdf]

Supplementary Materials

Modelling the effects of booster dose vaccination schedules and recommendations for public health immunization programs: the case of *Haemophilus influenzae* serotype b

Nadia A. Charania, Seyed M. Moghadas

The model

Model equations for transmission dynamics of the disease in the population with vaccination.

$$S' = \underbrace{(1-p)\mu N}_{\text{newborns without primary vaccination}} + \underbrace{(1-r)\kappa D}_{\text{loss of immunity}} + \underbrace{\omega P}_{\text{loss of immunity}} - \underbrace{\beta(I+\delta C)\frac{S}{N}}_{\text{infection of susceptibles}} - \underbrace{\mu S}_{\text{natural death}}$$

$$V_n' = \underbrace{p\mu N}_{\text{newborns with primary vaccination}} - \underbrace{\eta\beta(I+\delta C)\frac{V_n}{N}}_{\text{infection during partial protection}} - \underbrace{\sigma V_n}_{\text{aging to second year of life}} - \underbrace{\mu V_n}_{\text{natural death}}$$

$$V_p' = \underbrace{\sigma V_n}_{\text{aging to second year of life}} - \underbrace{\eta\beta(I+\delta C)\frac{V_p}{N}}_{\text{infection during partial protection}} - \underbrace{\phi V_p}_{\text{aging}} - \underbrace{\mu V_p}_{\text{natural death}}$$

$$V_b' = \underbrace{\alpha\phi V_p}_{\text{receiving booster during second year of life}} + \underbrace{r\kappa D}_{\text{receiving booster with deferral}} - \underbrace{\xi V_b}_{\text{loss of full protection}} - \underbrace{\mu V_b}_{\text{natural death}}$$

$$V_{bp}' = \underbrace{\xi V_b}_{\text{partial protection}} + \underbrace{\varepsilon R}_{\text{partial protection from infection}} - \underbrace{\pi\beta(I+\delta C)\frac{V_{bp}}{N}}_{\text{infection during partial protection}} - \underbrace{\tau V_{bp}}_{\text{loss of immunity}} - \underbrace{\mu V_{bp}}_{\text{natural death}}$$

$$D' = \underbrace{(1-\alpha)\phi V_p}_{\text{deferral of booster}} - \underbrace{\pi\beta(I+\delta C)\frac{D}{N}}_{\text{infection during partial protection}} - \underbrace{\kappa D}_{\text{loss of immunity}} - \underbrace{\mu D}_{\text{natural death}}$$

$$P' = \underbrace{\tau V_{bp}}_{\text{partial protection}} - \underbrace{\pi\beta(I+\delta C)\frac{P}{N}}_{\text{infection during partial protection}} - \underbrace{\omega P}_{\text{loss of immunity}} - \underbrace{\mu P}_{\text{natural death}}$$

$$L' = \underbrace{\beta(I+\delta C)\frac{S}{N}}_{\text{infection of susceptibles}} - \underbrace{\theta L}_{\text{developing infection}} - \underbrace{\mu L}_{\text{natural death}}$$

$$L_p' = \underbrace{\beta(I+\delta C)\frac{\eta(V_n+V_p+V_{bp})+\pi(D+P)}{N}}_{\text{infection during partial protection}} - \underbrace{\theta L_p}_{\text{developing infection}} - \underbrace{\mu L_p}_{\text{natural death}}$$

$$C' = \underbrace{q\theta L}_{\text{developing carriage}} + \underbrace{\theta L_p}_{\text{developing carriage}} - \underbrace{\gamma_1 C}_{\text{recovery from carriage}} - \underbrace{\mu C}_{\text{natural death}}$$

$$I' = \underbrace{(1-q)\theta L}_{\text{developing symptomatic infection}} - \underbrace{\gamma_2 I}_{\text{recovery from symptomatic infection}} - \underbrace{\mu I}_{\text{natural death}}$$

$$R' = \underbrace{\gamma_1 C}_{\text{recovery from carriage}} + \underbrace{\gamma_2 I}_{\text{recovery from symptomatic infection}} - \underbrace{\varepsilon R}_{\text{loss of full protection}} - \underbrace{\mu R}_{\text{natural death}}$$

Sensitivity and uncertainty analyses

To account for the uncertainty in the parameter space, we carried out a sensitivity analysis using the Latin Hypercube Sampling (LHS) technique [1] and calculated Partial Rank Correlation Coefficients (PRCC) to investigate the effect of parameter changes on the model outcomes, specifically on the number of Hib carriage. For this analysis, we considered nine parameters and their associated ranges, including η, π, q, γ_1, ξ, τ, ω, ε, δ, κ. To allow for the simultaneous variations of these parameters, samples of size 500 were generated in which each parameter was treated as a random variable and assigned a probability function. These parameters were uniformly distributed and sampled within their respective ranges. To calculate the PRCC, we considered the equilibrium state of the deterministic model structure for carriage infection as the response (model output), assuming that there is no correlation between the input parameters [2]. The parameters with large PRCC values (close to 1 or -1) and their corresponding p-values smaller than the significance level (0.05) have the largest influence on the model outcomes [3]. In this analysis, the transmission rate was calculated based on the sampled parameter values with fixed $R_0 = 1.4$. We examined scatter plots to verify the existence of monotonic relationships between the parameters used in the LHS and the response. The PRCC values are presented in Table 2. In the absence of booster, our analysis reveals that the rate of loss of immunity (ω) has the largest impact on the response. When booster vaccination was implemented, the rate of loss of full protection (ξ) was the most important parameter. The second most important parameters that affect the response are the rate of recovery from carriage (γ_1) and the reduction of susceptibility (π) during partial protection after vaccination or recovery from infection. Other parameters with p-value<0.001 had lower impact on the response. Considering PRCC values in Table 2, the results of our simulations remained unchanged in their qualitative behaviour for different reproduction numbers.

Table 2. Partial rank correlation coefficients (PRCC), and p-values of simulated parameters using the Latin Hypercube Sampling (LHS) technique.

Scenarios	Parameters and Partial Rank Correlation Coefficients (PRCC)									
	η	π	q	γ_1	ξ	τ	ω	ε	δ	κ
$\alpha = 0$, $r = 0$	0.273	0.485	0.118	0.628	–	-0.034	0.814	0.063	0.316	0.686
p-value	<0.001	<0.001	0.008	<0.001	–	0.45	<0.001	0.16	<0.001	<0.001
$\alpha = 1$, $r = 0$	0.330	0.442	0.084	0.536	-0.980	0.471	0.843	0.035	0.275	0.631
p-value	<0.001	<0.001	0.06	<0.001	<0.001	<0.001	<0.001	0.43	<0.001	<0.001
$\alpha = 0$, $r = 1$	0.321	0.556	0.119	0.461	-0.980	0.442	0.840	0.064	0.237	0.657
p-value	<0.001	0.008	<0.001	<0.001	<0.001	<0.001	<0.001	0.16	<0.001	<0.001

Reference

1. McKay M, Conover W, Beckman R. A comparison of three methods for selecting values of input variables in the analysis of output from a computer code. Technometrics. 1979;21(2):239–245.
2. Marino S, Hogue IB, Ray CJ, Kirschner DE. A methodology for performing global uncertainty and sensitivity analysis in systems biology. J Theor Biol. 2008;254(1):178–196.
3. Taylor R. Interpretation of the correlation coefficient: a basic review. J Diagn Med Sonogr. 1990;6(1):35–39.
